# On the Barometer

**Published:** 1811-01

**Authors:** Richard Walker


### *On the Barometer. By* Richard Walker, *Esq.* (Phil. Magazine.)

WATER exists in the atmosphere in two different states, viz. 1st, in a state of *chemical combination;* that is, so completely incorporated with the air, as to form with it one homogeneous transparent fluid:—and, 2dly, in a state of *mechanical combination;* which is, when the minute particles of water are merely suspended in the air, forming that state of the atmosphere, which is denominated cloudy or misty.

The dense state of the air being fittest for the *chemical combination* above mentioned; clear, dry weather, generally speaking, accompanies the higher degrees of the mercury in the barometer, whilst, a rare state of the air being less capable of receiving the water into chemical combination, it is then merely suspended in a state of *mechanical combination*, forming clouds, mists, &c.

Hence it follows, that, when the mercury stands at or near FAIR, clear dry weather is indicated *generally;* and when at or near RAIN, cloudy or wet weather; and when fluctuating mid-way, changeable weather.

It occasionally happens, however, that the atmosphere is cloudy, and even wet, whilst the barometer is as high as FAIR; and clear and dry, whilst the barometer stands as low as RAIN. The reason of this, in the first instance, is, that the air, having become replete or over-loaded with water, is incapable (by an alteration of temperature, viz. the air and its contents having become colder) of retaining or suspending it in a state of *chemical combination;* and in the latter case, which happens after rain, succeeding a continued dry state of the atmosphere, which having swept down the vapour with it in its descent; the air, though then in a rarer state, is yet sufficient to retain the proportion of water, now much reduced in quantity, in a state of chemical combination.

The particular or more immediate indication of the weather which is coming, arises from the alteration which is *taking place* in the density of the atmosphere, and which the barometer exhibits by the *rising or sinking state* of the mercury; the weather becoming comparatively *clearer* as the atmosphere is becoming denser, and *duller* as the atmosphere is becoming rarer*.

* The difference that might be supposed to arise in the height of the barometer from the effects of different degrees of heat on the atmosphere, may in observations of this nature be entirely disregarded, these effects being very nearly equalized by the expansion and contraction of the mercury in the barometer, from the same cause.

Hence,

Hence, if the barometer were as portable and as convenient for reference as a watch, we should seldom be at a loss to know, at least for short intervals, what kind of weather was coming*.

The ordinary range of the barometer in this climate is from RAIN to FAIR; rising however, occasionally, as high as SETTLED FAIR; and sometimes, though very rarely, as high as VERY DRY: and sinking, occasionally, as low as MUCH RAIN; and sometimes, though very rarely, as low as STORMY.

It is scarcely necessary to observe that north and east winds, in consequence of passing to us from a colder climate, and over land, bring a denser, colder, and dryer atmosphere; and south and west winds, coming to us from a warmer climate, and over the sea, bringing a rarer, warmer, and damper atmosphere; and moreover, that the capacity of air for retaining water in a state of chemical combination, is *increased* by coming from a colder to a warmer temperature; and *diminished*, by coming from a warmer to a colder temperature.

It must be equally apparent, that the greater or less elevation of the clouds depends upon their own degree of density, and that of the atmosphere which supports them.

With regard to the immediate causes of the direction and changes of the wind in this climate, I consider them as involved in too much obscurity and uncertainty to say any thing satisfactorily about them; and with respect to electricity, which though doubtless a powerful agent in meteorological effects, I consider it rather as a matter of curious speculation than of practical utility.

I have therefore only to add, that by a due consideration of the causes enumerated above, connected with the more obvious effects of the sun's varying influence in raising and dispelling vapours, we may, I think, account pretty satisfactorily for the various vicissitudes of weather, which mark the different seasons throughout the year; and by the relation of the barometer to those causes, be enabled to foresee, with a considerably greater degree of certainty than is commonly supposed, the different changes of weather which are, at all times, about to take place.

---

* As the atmosphere is almost constantly varying in its degree of density; so is the barometer, which is an accurate measure of its density, as constantly varying in its altitude, and should therefore be frequently referred to.

